# Bihemispheric alterations in myelination in children following unilateral perinatal stroke

**DOI:** 10.1016/j.nicl.2018.06.028

**Published:** 2018-06-27

**Authors:** Sabrina Yu, Helen L. Carlson, Aleksandra Mineyko, Brian L. Brooks, Andrea Kuczynski, Jacquie Hodge, Sean Dukelow, Adam Kirton

**Affiliations:** a Calgary Pediatric Stroke Program, University of Calgary, Calgary, AB, Canada; b Department of Pediatrics, Cumming School of Medicine, University of Calgary, Calgary, AB, Canada; c Department of Clinical Neurosciences, Cumming School of Medicine, University of Calgary, Calgary, AB, Canada; d Alberta Children's Hospital Research Institute, University of Calgary, Calgary, AB, Canada; e Hotchkiss Brain Institute, University of Calgary, Calgary, AB, Canada

**Keywords:** Perinatal stroke, Myelination, White matter, Developmental plasticity, Cerebral palsy

## Abstract

**Background:**

Stroke is a leading cause of perinatal brain injury with variable outcomes including cerebral palsy and epilepsy. The biological processes that underlie these heterogeneous outcomes are poorly understood. Alterations in developmental myelination are recognized as a major determinant of outcome in preterm brain injury but have not been explored in perinatal stroke. We aimed to characterize myelination in hemiparetic children after arterial perinatal stroke, hypothesizing that ipsilesional myelination would be impaired, the degree of which would correlate with poor outcome.

**Methods:**

Retrospective, controlled cohort study. Participants were identified through the Alberta Perinatal Stroke Project (APSP), a population-based research cohort (n > 400). Inclusion criteria were: 1) MRI-confirmed, unilateral arterial perinatal stroke, 2) T1-weighted MRI after 6 months of age, 3) absence of other neurological disorders, 4) neurological outcome that included at least one of the following tests - Pediatric Stroke Outcome Measure (PSOM), Assisting Hand Assessment (AHA), Melbourne Assessment (MA), neuropsychological evaluation (NPE), and robotic sensorimotor measurements. FreeSurfer software measured hemispheric asymmetry in myelination intensity (primary outcome). A second method using ImageJ software validated the detection of myelination asymmetry. A repeated measures ANOVA was used to compare perilesional, ipsilesional remote, and contralesional homologous region myelination between stroke cases and typically developing controls. Myelination metrics were compared to clinical outcome measures (*t*-test, Pearson's correlation).

**Results:**

Twenty youth with arterial stroke (mean age: 13.4 ± 4.2yo) and 27 typically developing controls (mean age: 12.5 ± 3.7yo) were studied in FreeSurfer. Participants with stroke demonstrated lower myelination in the ipsilesional hemisphere (p < 0.0001). Myelination in perilesional regions had lower intensity compared to ipsilesional remote areas (p < .00001) and contralesional homologous areas (p < 0.00001). Ipsilesional remote regions had decreased myelination compared to homologous regions on the contralesional hemisphere (p = 0.016). Contralesional myelination was decreased compared to controls (p < 0.00001). Myelination metrics were not strongly associated with clinical motor, robotic sensorimotor, or neuropsychological outcomes though some complex tests requiring speeded responses had moderate effect sizes.

**Conclusion:**

Myelination of apparently uninjured brain in both the ipsilesional and contralesional hemispheres is decreased after perinatal stroke. Differences appear to radiate outward from the lesion. Further study is needed to determine clinical significance.

## Highlights

• Myelination is altered in the lesioned hemisphere after perinatal stroke.
• The uninjured, contralesional hemisphere also demonstrates differences in myelination.
• Simple software can estimate MRI myelination abnormalities in children with perinatal brain injury.

## 1 Introduction

Stroke is a leading cause of perinatal brain injury, cerebral palsy, and lifelong disability (Kirton and deVeber, 2013). With an incidence of at least 1 per 2500 live births, there are currently 10,000 Canadian children living with perinatal stroke (Agrawal et al., 2009; Mineyko and Kirton, 2011). Modern clinicoradiographic criteria facilitate the diagnosis of precise perinatal stroke diseases. Perinatal arterial ischemic strokes (AIS) are focal brain injuries secondary to cerebral artery occlusion occurring between 20 weeks gestation and 28th post-natal day. AIS can present acutely within the first month of life (neonatal arterial ischemic stroke; NAIS) or can be diagnosed retrospectively as arterial presumed perinatal ischemic stroke (APPIS) later in childhood (Mineyko and Kirton, 2011). Additional perinatal stroke diseases include fetal periventricular venous infarction (Kirton et al., 2008); cerebral sinovenous thrombosis (deVeber et al., 2001), and neonatal hemorrhagic stroke (Cole et al., 2017).

Despite relatively homogenous lesions incurred at precise time points early in development, outcomes following perinatal stroke vary widely. Approximately 60% of children have cerebral palsy, 30–60% experience epilepsy, 25% show language delay, and at least 20–30% manifest behavioural abnormalities (Kirton and deVeber, 2013; Lee et al., 2005). These neurological outcomes are complex and inter-related, such as the association of epilepsy with worse cognitive and developmental outcomes in perinatal stroke (Mineyko et al., 2017; van Buuren et al., 2013). Understanding the differences in how the brain develops following such early injuries is key to advancing new therapeutic interventions. Fortunately, combinations of animal (Martin et al., 2007) and human (Eyre, 2007; Kirton, 2013; Staudt, 2007) evidence are informing increasingly accurate models of motor system organization following perinatal stroke. For example, the role of the contralesional primary motor cortex in controlling a hemiparetic limb via preserved ipsilateral corticospinal projections appears to be an important determinant of clinical function (Zewdie et al., 2016). Such models are clinically relevant, identifying cortical targets for neuromodulation that have recently been translated into multiple clinical trials of non-invasive neurostimulation that suggest efficacy in enhancing function in hemiparetic children (Gillick et al., 2014; Kirton et al., 2016a, Kirton et al., 2016b; Kirton et al., 2017). Although imaging has been key in defining these valuable models, one component that remains to be explored is myelination.

Alterations in myelination are recognized as a major determinant of outcome in preterm brain injury (Aylward, 2014; Back and Miller, 2014) but are unexplored in perinatal stroke. Myelin controls the speed of impulse conduction through axons, and is critical for virtually all brain functions. The organization of white matter tracts and thickness of myelin sheaths are essential in the cognitive, behavioural, and emotional development of children (Barnea-Goraly et al., 2005; van der Knaap et al., 1991). White matter connectivity has been linked with clinical outcome in cerebral palsy, including perinatal stroke. Diffusion imaging of the corticospinal tracts has demonstrated associations between motor pathway structural connectivity and clinical function in perinatal stroke (Hodge et al., 2017; van der Aa et al., 2011). We have also recently shown that both motor (Kuczynski et al., 2016) and sensory (Kuczynski et al., 2017a) tractography measures of white matter connectivity correlate with detailed robotic measures of clinical function after perinatal stroke. These studies suggest the importance of white matter health in perinatal stroke outcome but are limited to tract-specific analyses and do not explore myelination itself.

Intensity of white matter signal on T1 MRI is an established imaging biomarker of brain myelination validated by histopathological studies (Oishi et al., 2013; Stüber et al., 2014). Intensity of white matter signal on T1 MRI is an established imaging biomarker of brain myelination validated by histopathological studies (Oishi et al., 2013; Stüber et al., 2014). The extent and distribution of myelin-bound cholesterol can be inferred by the brightness of white matter on a T1 map (Ganzetti et al., 2014; Koenig, 1991). Although T1-weighted MRI is typically used to qualitatively inform on myelin, the fast scanning times and non-invasive nature make it potentially well-suited to quantitatively assess myelination in clinical investigations. Such alternative approaches to exploring myelination may provide an improved understanding of developmental plasticity but have not been applied to the perinatal stroke population.

We performed a retrospective, controlled cohort study to quantify myelination across both hemispheres in children with arterial perinatal stroke. We hypothesized that ipsilesional myelination would be impaired, the degree of which would correlate with poor neurological outcome.

## 2 Methods

### 2.1 Participants

Participants with AIS were identified through the Alberta Perinatal Stroke Project (APSP), a population-based research cohort of >400 affected children (Cole et al., 2017). Inclusion criteria were: 1) MRI-confirmed, unilateral arterial perinatal stroke, 2) T1-weighted MRI > 6 months of age, 3) absence of other neurological disorders, 4) neurological assessment that included at least one of the following tests: Pediatric Stroke Outcome Measure (PSOM), Assisting Hand Assessment (AHA), Melbourne Assessment (MA), robotic assessment of sensorimotor function, and structured neuropsychological evaluation (NPE) at >2 years of age, and 5) informed consent/assent.

Typically developing children were recruited from the general public and an established community healthy controls program (www.hiccupkids.ca). Control participants were right handed and had no neurodevelopmental or psychiatric conditions and no contraindications to MRI. Methods were approved by the institutional research ethics board.

### 2.2 Neuroimaging

**Fig. 1:**
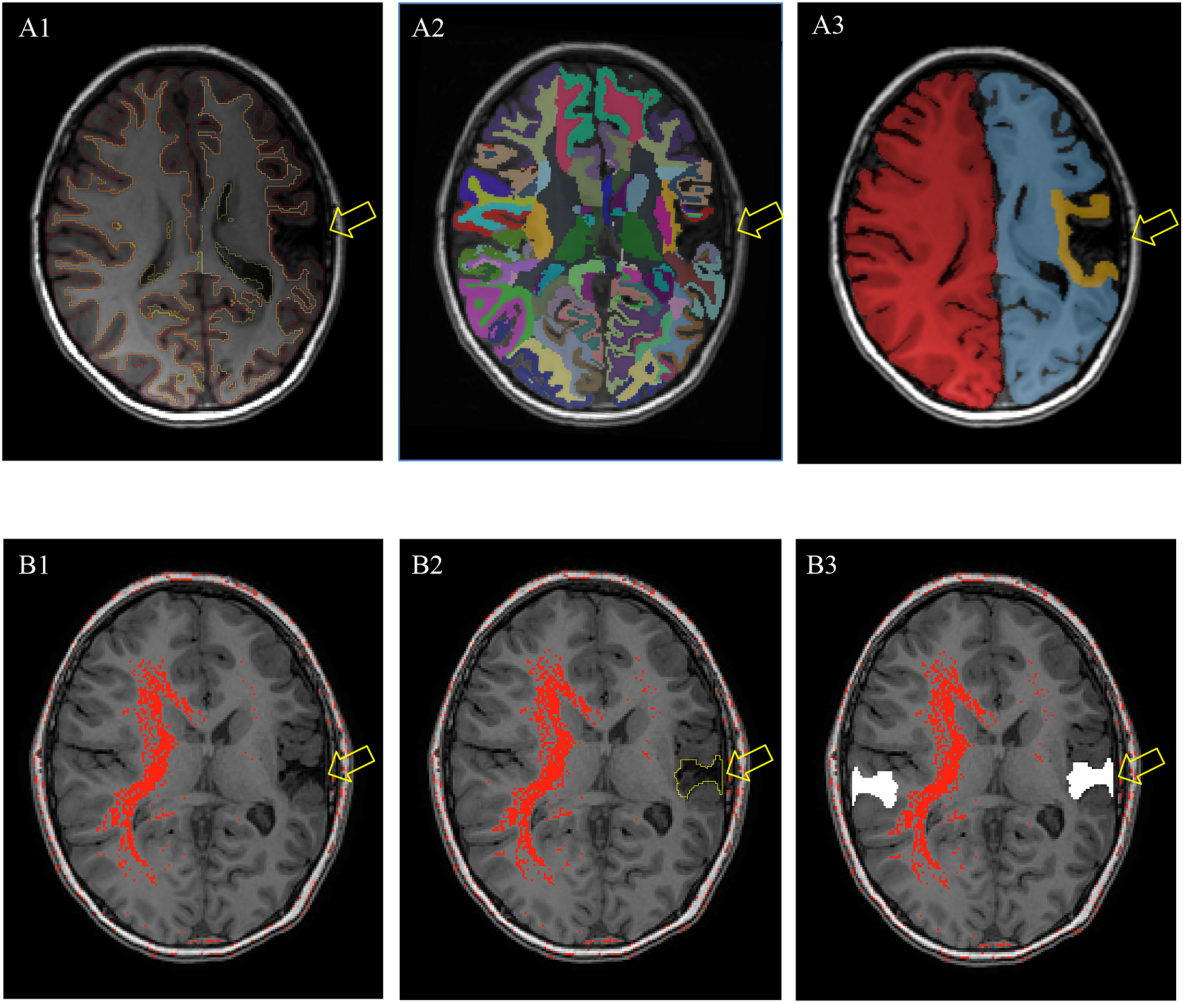
Imaging methods. (A) The *FreeSurfer* method performed semi-automated segmentation with parcellation of the cerebral cortex based on the structure of gyri and sulci (A1), subcortical segmentation (A2), and further classification into perilesional (yellow), ipsilesional remote (blue), or contralesional regions (purple) to measure segmental white matter intensity (A3). (B) The *ImageJ* thresholding technique adjusted the threshold tool upwards until positive pixels first appeared in white matter regions bilaterally. The intensity value immediately below this was then used as a cut-off, with any remaining positive pixels measured as the hemispheric difference in myelination intensity (B1). Stroke lesion manually traced (B2), and excluded for intensity measurements of comparable areas between hemispheres (B3). Any remaining positive (greater T1 intensity) pixels in the homologous, contralateral white matter were then scored to represent the difference. Lesion indicated by arrow.

Images were obtained from two scanners and analyzed accordingly. *FreeSurfer* requires higher resolution images from research scans for best processing and parcellation results. A larger group of clinically obtained scans was also included for the *ImageJ* analysis which is less dependent on image quality. Research scans were acquired for a subset of participants using the Alberta Children's Hospital Research 3.0 Tesla GE MR750w MRI scanner (GE Healthcare, Waukesha, WI) with an MR Instruments (Minnetonka, MN) 32-channel receive-only head coil. T1-weighted anatomical fast spoiled gradient echo (FSPGR) images were acquired in the axial plane [voxel size = 1.0 mm^3^ isotropic; repetition time (TR) = 8.5 ms; echo time (TE) = 3.2 ms; field of view (FOV) = 256x166mm] (Kuczynski et al., 2017a). T1-weighted images were reconstructed and analyzed using FreeSurfer imaging software (http://surfer.nmr.mgh.harvard.edu) (see Fig. 1A). The technical details of FreeSurfer processing are described previously (Desikan et al., 2006; Fischl et al., 2002). Ghosh et al. (2010) has demonstrated comparisons of morphology in children to be accurate and feasible. Briefly, this processing includes automated removal of non-brain tissue, automated Talairach transformation of individual MRI scans into a 3-D coordinate system to map structures independent from individual differences in the size and overall shape of the brain, and segmentation of the subcortical structures (Fischl et al., 2002). Additional processing and analysis can then be performed, including cortical parcellation based on the structure of gyri and sulci (Desikan et al., 2006) (Fig. 1). Greater accuracy in mapping tissue structures and borders has been demonstrated with these procedures than with volumetric registration (Fischl et al., 2008).

From the white matter parcellation, regions were further manually classified into perilesional regions, ipsilesional remote regions, and contralesional homologous regions. Since the classification of brain regions may be unreliable due to potentially large deformations after stroke, manual reclassification into these three regions was needed to determine intra-hemispheric differences in myelination intensity. Perilesional regions included any segmented areas that were directly touching the stroke lesion, with the distal border defined by the automatic segmentations. Ipsilesional remote regions were those not touching the lesion. Contralesional homologous regions included areas that corresponded to ipsilesional remote regions on the contralateral hemisphere. *Freesurfer* morphometric procedures have been demonstrated to show good test-retest reliability across scanner manufacturers and other imaging parameters (Jovicich et al., 2009). The automated intensity correction procedures alter the voxel values, so they were not applied to preserve original tissue intensity values (Fischl et al., 2002). To minimize intensity inhomogeneity caused by B1 and coil profiles, white matter intensity was compared to the contralateral hemisphere within each participant. Hemispheric asymmetry in myelination intensity (primary outcome) was quantified in *FreeSurfer*. Asymmetry was calculated as (contralateral intensity)/(ipsilesional intensity): the ratio between image intensity values from contralesional hemisphere versus the ipsilesional hemisphere. This assumed the contralesional white matter intensity was normal (see results below). Asymmetry values >1 indicate higher contralesional WM intensity values, implying damage to the ipsilesional WM. Asymmetry values were also calculated for remote regions of the lesioned hemisphere and the contralateral homologue.

Clinical scans were acquired using a Siemens Avanto 1.5 Tesla MRI scanner (Siemens, Erlangen, Germany). Spin-echo T1-weighted images were acquired in the axial plane [voxel size = 1.0 mm in-plane; slice thickness = 5 mm; repetition time (TR) = 580 ms; echo time (TE) = 15 ms; field of view (FOV) = 192 × 256mm). These images were analyzed using *ImageJ* freeware (https://imagej.nih.gov/ij/) (see Fig. 1B). The thresholding tool scores each pixel as positive or negative on a scale of 1 to 255 degrees of brightness. T1 images were imported and analyzed starting from the most inferior slice where white matter was visible in both hemispheres. Subsequent superior slices were examined sequentially, and the threshold adjusted accordingly. The upper pixel intensity threshold was adjusted upwards until positive pixels first appeared in white matter regions bilaterally. Assuming the contralesional hemisphere was normal, the intensity value immediately below this was then used as a cut-off, with any remaining positive pixels measured as the hemispheric difference in myelination intensity. Thus, values approaching zero indicate no myelination asymmetry between hemispheres, and values greater than zero indicate the presence of asymmetry. This thresholding technique had previously been used to quantify descending corticospinal tract and remote diffusion-weighted imaging signal changes in newborns and children with arterial ischemic stroke (Domi et al., 2009; Kirton et al., 2016a, Kirton et al., 2016b; Kirton et al., 2007b). The stroke lesion was manually traced and excluded from intensity scoring to measure comparable areas in both hemispheres. Asymmetry in myelination intensity was reported as the difference between contralesional and ipsilesional intensity values (Fig. 1B). Myelination intensity, infarct volume, and total brain volume were measured in every slice using the thresholding tool. The same reader blindly re-analyzed a randomly selected subset (n = 10) 2 weeks later to assess intra-rater reliability in *ImageJ*.

### 2.3 Sensorimotor assessments

Upper extremity motor function was assessed using the Assisting Hand Assessment [AHA] and Melbourne Assessment of Upper Limb Function [MA]. Both are evidence-based, validated measures of motor function that were administered by experienced pediatric occupational therapists who were blinded to other clinical and imaging data. For all motor functioning tasks, higher scores indicate better performance. The AHA is a 22-item assessment tool that measures spontaneous bi-manual hand function in children with motor impairments (Krumlinde-Sundholm et al., 2007). It has the advantage of simulating real-world activities to measure spontaneous bi-manual motor function rather than testing the affected hand in isolation. Motor outcomes were quantified and converted to AHA logit units (log-odds probability units) ranging from 0 (affected hand not used at all) to 100 (normal). The MA consists of 16 tasks that measure unilateral motor function in hemiparetic upper limbs (Bourke-Taylor, 2003). Typical tasks include reaching and grasping different sized objects reflecting finger dexterity and speed of motion. Raw scores range from 0 (no achievement of tasks) to 122 points (normal), and are expressed as percentages [MA = (# of points achieved/total # points possible)*100].

A subset of participants were assessed with the kinesiological instrument for normal and altered reaching movements (KINARM) robot (Kuczynski et al., 2017b). Participants completed position-matching and kinesthesia tasks first without (NV) and then with vision (V). The following outcomes were assessed for position-matching: (1) Variability (Var_xy_) of hand position: a measure of variability between the spatial location of the affected hand (moved by the robot) versus the patient-matched position of the unaffected hand (in cm); (2) Spatial shift (Shift_xy_): a measure of the spatial translation of the workspace (in cm); and (3) Expansion/Contraction Area (Area_xy_) of the workspace: a measure of the degree of contraction or expansion of the workspace matched by the unaffected arm (in cm) (Kuczynski et al., 2016). Outcome parameters assessed in the kinesthesia task were: (1) Response latency (RL): the difference in movement onset between the active/passive arms (in milliseconds); (2) Initial direction error (IDE): the angular deviation between the active/passive arms at peak hand speed (in degrees); (3) Peak speed ratio (PSR): the ratio of peak hand speed of the active versus passive arm; (4) Path length ratio (PLR): the ratio of the distance travelled by the active arm versus passive arm. The variability in each of these four measures (denoted by a “v” at the end of each parameter name) was computed using the standard deviation across all trials (Kuczynski et al., 2017b). Details on the calculation of outcome parameters are described elsewhere (Dukelow et al., 2010; Semrau et al., 2013).

### 2.4 Neuropsychological outcomes

The Pediatric Stroke Outcome Measure (PSOM) is a validated tool consisting of a 115-item neurological examination (Kitchen et al., 2012). It measures neurological deficits and function across 5 subscales: right sensorimotor, left sensorimotor, language production, language comprehension, and cognitive/behavioural. To measure cognitive outcomes after perinatal stroke, the non-motor PSOM score was calculated as the sum of the language production, language comprehension, and cognitive/behavioural subscores, ranging from 0 (no deficit) to 6 (maximum score of 2 in all 3 domains). Non-motor PSOM scores were dichotomized as poor if the total was >1 (moderate to severe deficits).

For a subset of participants, formal neuropsychological evaluations were performed by a clinical neuropsychologist using test versions appropriate for the child's age and concerns on referral as described previously (Mineyko et al., 2017). Domains of interest included reasoning, visual-construction, verbal learning and memory, executive functions, and behaviour. Percentile scores based on each test's population norms were used as continuous dependent variables for each subset of tests. Neuropsychological tests that were selected for further analysis were the WISC-IV Full Scale IQ (FSIQ), WISC-IV Processing Speed Index (PSI), CVLT-C Total Trials 1-5, NEPSY-II Word Generation, NEPSY-II Inhibition Naming Time, NEPSY-II Inhibition Switching, BRIEF-P GEC, and ADHD Parent Report Total. Because this study was a retrospective investigation utilizing clinical assessments, not all WISC-IV subtests were available for all participants and as such, FSIQ was estimated from available subtests (Vocabulary and Matrix Reasoning) as per Sattler (2001).

Sleep electroencephalogram (EEG) reports were reviewed when available to explore associations with myelination measures. Continuous spikes and wave in sleep (CSWS), a form of epileptic encephalopathy, was interpreted as spike and wave discharges occurring in >75% of slow wave sleep. EEG reports were independently classified as CSWS or non-CSWS by a blinded epileptologist as described previously in this population (Mineyko et al., 2017).

### 2.5 Statistical analyses

Following confirmation of a normal distribution, a mixed design repeated measures ANOVA was used to compare perilesional, ipsilesional remote, and contralesional homologous regions of myelination estimates across groups and location. Myelination ratios for stroke cases were compared to typically developing controls (*t*-test), dichotomized PSOM scores (t-test), motor assessments (Pearson's correlation), neuropsychology tests (Spearman's correlation), and EEG classification (t-test). A partial Spearman's correlation was used to compare sensory function with correction for age, total brain volume, and multiple comparisons. Pearson's correlation test was used to validate *FreeSurfer* findings with the *ImageJ* results. Bonferroni correction was applied for consideration of multiple comparisons. Intraclass correlation was used to assess intra-rater reliability for manual thresholding technique in *ImageJ*. SPSS 24.0 was used to perform the statistical analyses.

## 3 Results

### 3.1 Population

Thirty-nine participants with AIS (range 0.5–18.0 years) were initially included for *ImageJ* analysis. Of this population, a subset of 26 had T1-weighted 3 T MRI imaging for *FreeSurfer* analysis. Six participants were subsequently excluded from *FreeSurfer* analysis due to reduced quality anatomical scans caused by excessive head motion or errors in modelling. For final analysis in *FreeSurfer*, 20 children with AIS (median age 12.4 years, range: 6.8–18.8 years, 60.0% male) and 27 typically developing children (median age 12.0 years, range: 6.0–19.0 years, 55.6% male) were included. None of these were excluded from *ImageJ* analysis, which was less dependent on image quality. All 20 participants in *FreeSurfer* analysis were included in the larger *ImageJ* analysis population. Group demographic information is displayed in Table 1.

**Table 1:** Population demographics.

Category	*FreeSurfer* Analysis		*ImageJ* Analysis	
	AIS Freesurfer (*n* = 20)	TDC (*n* = 27)	AIS ImageJ (*n* = 39)	TDC (n = 15)
Age: Mean years (SD) [range]	13.4 (4.2) [6.6–18.8]	12.5 (3.7) [6.0–19.0]	7.9 (5.0) [0.5–18.0]	12.3 (4.1) [6.0–18.0]
Sex [%]				
Male	N = 12 [60.0%]	*N* = 15 [55.6%]	*N* = 21 [53.8%]	*N* = 10 [66.7%]
Female	*N* = 8 [40.0%]	*N* = 12 [44.4%]	*N* = 18 [46.2%]	*N* = 5 [33.3%]
Total	*N* = 20	*N* = 27	*N* = 39	N = 15
Outcomes: Mean [range]				
AHA	63.2 [35–100]	–	58.0 [33−100]	–
MA	75.3 [41.6–100]	–	69.7 [32.6–100]	–
PSOM	52.6% poor	–	70.6% poor	–
NPE		–		–
WISC-IV FSIQ	18.4 [0.4–79]	–	16.3 [0.1–79]	–
WISC-IV PSI	11.8 [0.1–50]	–	13.2 [0.1–50]	–
CVLT-C	62.9 [2–95]	–	47.9 [0.1–97]	–
NEPSYII WdGen Semantic	49.3 [0.1–75]	–	34.8 [0.1–75]	–
NEPSYII Inh Naming	31.5 [2–91]	–	29.3 [0.1–91]	–
NEPSYII Inh Switch	24.2 [0.1–99]	–	31.1 [0.1–100]	–
BRIEF-P GEC	78.4 [32−100]	–	78.3 [8–100]	–
ADHD Parent Total	72.4 [25–99]	–	65.0 [17–99]	–
EEG	–	–	17.6% CSWS	–

BRIEF-P and ADHD scales are negatively scored, i.e., higher scores represent more problems. *BRIEF-P percentiles were calculated based on BRIEF-P T-scores.

Acronyms and Abbreviations: AHA: Assisting Hand Assessment; MA: Melbourne Assessment; PSOM: Pediatric Stroke Outcome Measure; WISC-IV: Wechsler Intelligence Scale for Children 4th edition; PSI: Processing Speed Index; CVLT-C: California Verbal Learning Test Children's Edition; NEPSY-II : A Developmental NEuroPSYchological Assessment 2nd edition; WdGen Semantic: Word Generation Semantic Total; Inh Naming: Inhibition Naming; Inh Switch: Inhibition Switching; BRIEF-P GEC: Behaviour Rating Inventory of Executive Function Preschool Version; ADHD: Attention Deficit Hyperactivity Disorder; EEG: Electroencephalogram.

### 3.2 Imaging measures of myelination

The *ImageJ* thresholding technique demonstrated high intra-rater reliability (p = 0.98). Neither subject sex (r = 0.01, p = 0.64) nor age (r = 0.01, p = 0.94) were associated with *ImageJ* myelination scores. In *FreeSurfer*, a higher degree of myelination asymmetry was found in females (p = 0.01). Myelination intensity was not correlated with age (r = −0.05, p = 0.84). *FreeSurfer* hemispheric myelination asymmetry correlated with *ImageJ* scores in stroke participants (r = 0.47, p = 0.049. For controls, *FreeSurfer* and *ImageJ* myelination ratios were not correlated (r = −0.003, p = 0.99).

### 3.3 FreeSurfer myelination

**Fig. 2:**
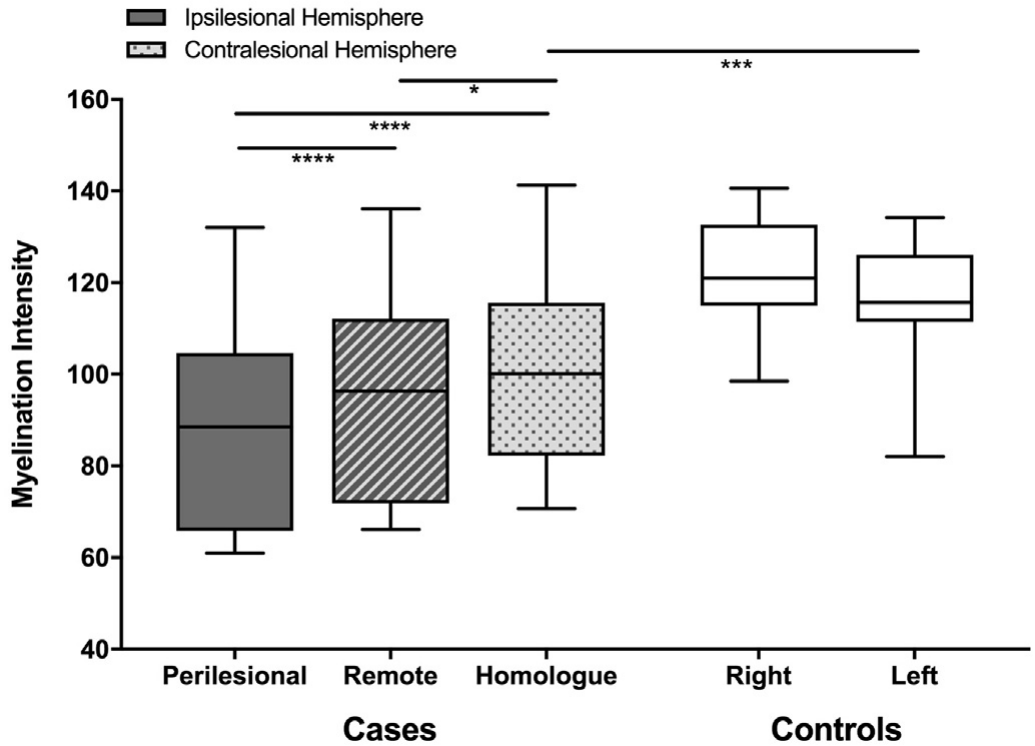
Regional myelination: *FreeSurfer* method. Myelination intensity was lowest in perilesional white matter, followed by remote regions in the lesioned hemisphere. Myelination intensity in homologous regions of the contralesional hemisphere were higher but still less than both hemispheres in typically developing controls where right hemisphere values were higher than left.

Myelination intensity across groups and locations is summarized in Fig. 2. Asymmetry in myelination intensity between hemispheres was observed in both cases and controls. In controls, the non-dominant (right) hemisphere displayed higher myelination intensities (right: mean = 122.24 ± 11.0, left: mean = 117.13 ± 11.6, p < 0.001). Compared to control ratios, stroke cases demonstrated a greater degree of asymmetry with lower myelination of all white matter in the ipsilesional hemisphere (p < 0.0001). Myelination intensity in perilesional regions of the stroke hemisphere (mean = 86.33 ± 21.0) was significantly lower than ipsilesional remote (p < 0.00001) and contralesional homologous areas (p < 0.00001). Myelination in ipsilesional remote areas of white matter were the next lowest (mean 94.06 ± 22.0), and significantly lower than contralesional homologues (mean 100.96 ± 18.2, p = 0.016). Differences in ipsilesional remote areas were even greater when compared to a mean value of myelination intensity in controls (p < 0.00001). Contralesional areas had lower than normal myelination intensity as compared to both hemispheres in controls (right hemisphere: p < 0.00001, left hemisphere: p = 0.0003).

**Fig. 3:**
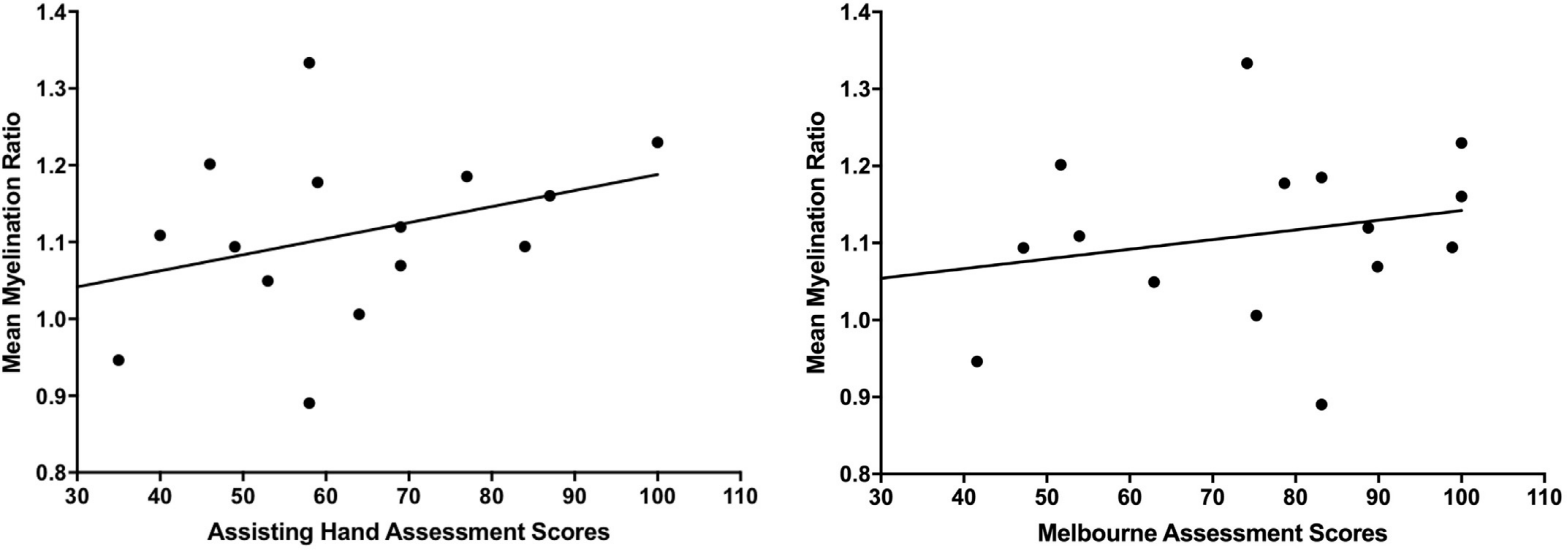
Myelination and motor outcome. Motor assessments were not related to *FreeSurfer* myelination ratios (AHA: p = 0.22, r = 0.33) (MA: p = 0.37, r = 0. 24).

**Fig. 4:**
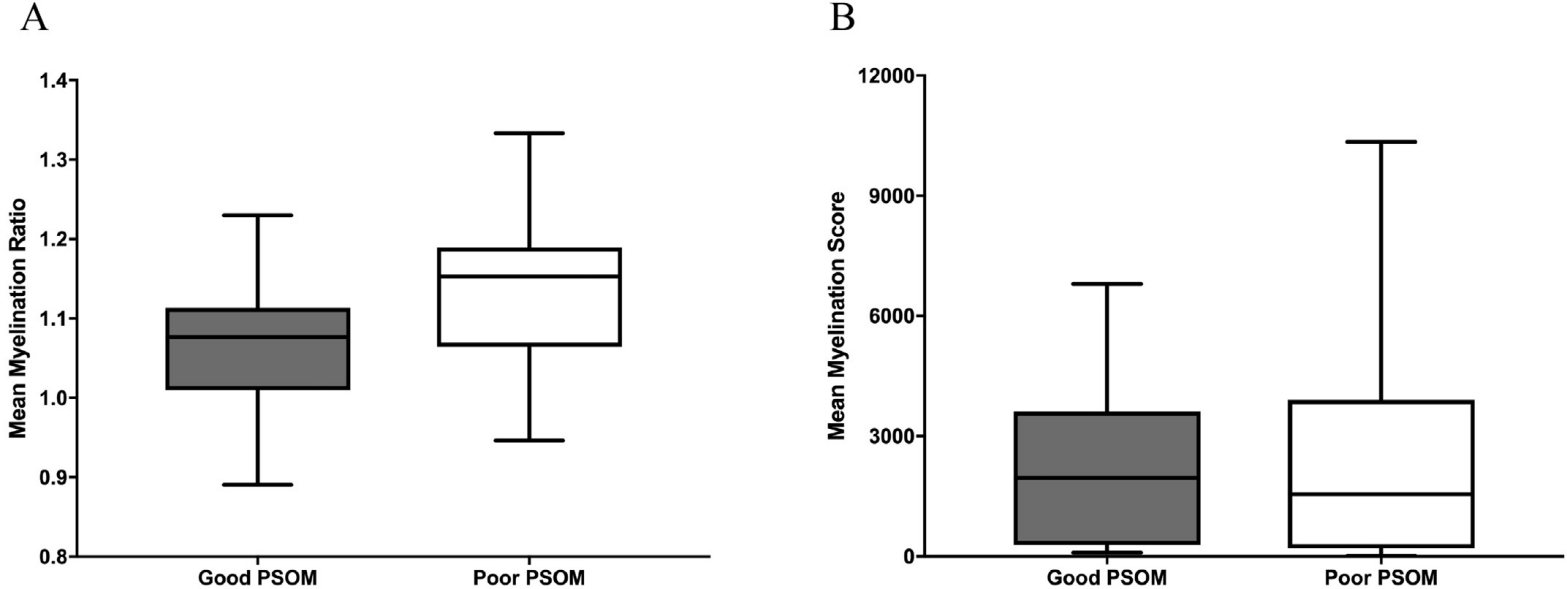
Myelination and stroke outcomes. (A) PSOM scores were not correlated with *FreeSurfer* myelination ratios (p = 0.07). (B) *ImageJ* myelination scores were not associated with PSOM scores (p = 0.99).

For the twenty participants with AIS analyzed with *FreeSurfer* software, fifteen had AHA and MA assessments (mean scores of 63.2 ± 18.2 and 75.3 ± 19.6 respectively). There was no correlation between myelination ratios and AHA (r = 0.33, p = 0.22) or MA scores (r = 0.24, p = 0.37) (Fig. 3). There were also no correlations observed between myelination ratios and robotic measures of sensorimotor function (see Table 2). Of the participants analyzed in *FreeSurfer*, non-motor PSOM scores were dichotomized into nine cases with good outcome and ten cases with poor (Fig. 4). Myelination ratios in participants with good PSOM scores (mean 1.07 ± 0.09) may have trended toward lower values than those with poor outcome (mean ratio 1.14 ± 0.10, p = 0.07). No neuropsychological outcomes were significantly associated with myelination intensity (see Table 2), however, the NEPSY-II Inhibition-Switching task, a measure of speeded executive function, had a small to moderate effect size (ρ = 0.33).

**Table 2:** Summary of statistical results for myelination ratios, cognitive and sensorimotor outcomes.

Category	AIS FreeSurfer (*n* = 20)	AIS ImageJ (n = 39)
AHA	r = 0.33, p = 0.22 (*n* = 15)	*r* = −0.30, *p* = 0.16 (*n* = 26)
MA	r = 0.24, p = 0.37 (n = 15)	*r* = −0.22, *p* = 0.31 (n = 26)
Non-Motor PSOM	*p* = 0.07 (*n* = 18)	*p* = 0.99 (*n* = 34)
NPE		
WISC-IV FSIQ	ρ =0.10, *p* = 0.79 (*n* = 10)	ρ = −0.35, *p* = 0.22 (*n* = 16)
WISC-IV PSI	ρ =0.20, *p* = 0.54 (n = 12)	ρ = −0.28, *p* = 0.25 (*n* = 31)
CVLT-C Total	ρ =0.09, p = 0.79 (*n* = 11)	ρ =0.40, *p* = 0.09 (*n* = 30)
NEPSYII WdGen Semantic	ρ = −0.05, *p* = 0.89 (n = 10)	ρ =0.08, *p* = 0.76 (n = 26)
NEPSY II Inh-Naming Comp Time	ρ = −0.21, *p* = 0.49 (*n* = 13)	ρ = −0.06, *p* = 0.81 (n = 30)
NEPSY II Inh-Switch Comp Time	ρ =0.33, *p* = 0.32 (*n* = 11)	ρ = −0.31, *p* = 0.28 (*n* = 25)
BRIEF-P GEC	ρ =0.12, *p* = 0.69 (n = 13)	ρ =0.18, *p* = 0.46 (n = 30)
ADHD Parent Total	ρ =0.24, *p* = 0.43 (n = 13)	ρ = −0.13, *p* = 0.60 (n = 31)
KINARM		
Var_xy_ (cm) NV	ρ = −0.00, *p* = 1.0 (*n* = 17)	–
Var_xy_ (cm) V	ρ = −0.23, *p* = 0.40 (*n* = 17)	–
IDE NV	ρ =0.30, *p* = 0.30 (n = 17)	–
IDE V	ρ =0.40, *p* = 0.15 (n = 17)	–
IDE_V_ NV	ρ =0.19, *p* = 0.52 (n = 17)	–
IDE_V_ V	ρ =0.16, *p* = 0.59 (n = 17)	–
RL NV	ρ =0.19, *p* = 0.53 (n = 17)	–
RL V	ρ = −0.33, p = 0.25 (n = 17)	–
RL_V_ NV	ρ = −0.05, *p* = 0.87 (n = 17)	–
RL_V_ V	ρ = −0.26, *p* = 0.36 (n = 17)	–

Acronyms: WISC-IV: Wechsler Intelligence Scale for Children 4th edition; CVLT-C: California Verbal Learning Test Children's Edition; NEPSY-II: A Developmental NEuroPSYchological Assessment 2nd edition; BRIEF-P: Behaviour Rating Inventory of Executive Function Preschool Version; ADHD: Attention Deficit Hyperactivity Disorder; IDE: Initial direction error in vision (V) and no vision (NV) conditions; RL: Response latency in vision (V) and no vision (NV) conditions.

### 3.4 ImageJ myelination

Findings of asymmetry in myelination intensity between hemispheres were confirmed with *ImageJ* in both cases and controls. All 39 AIS participants displayed myelination asymmetry (mean = 2613.78 ± 2731.6). In controls, the non-dominant (right) hemisphere again displayed higher myelination asymmetry (2677.55 ± 6854.5). Lesion size (as % of whole brain) was not correlated with *ImageJ* myelination score (p = 0.67). In *ImageJ*, the mean AHA and MA scores of participants were 58.4 ± 17.4 and 70.8 ± 20.8 respectively. Again, no correlations were observed between myelination ratios and AHA (r = −0.28, p = 0.16) or MA scores (r = −0.20, p = 0.33).

For *ImageJ* data, ten cases (29%) had good PSOM scores compared to twenty-four with poor. Myelination ratios were not associated with PSOM scores (p = 0.99). *ImageJ* measures of myelination were not significantly associated with neuropsychological subtests based on p-values alone (see Table 2), however, there were several with small to moderate effect sizes (ρ > 0.28) such as FSIQ, PSI, CVLT-C and NEPSY Inhibition-Switching.

Additionally, thirty-four participants with *ImageJ* had sleep EEG tests performed, of which six cases (18%) of CSWS were identified. No association between sleep-related epileptic encephalopathy and ipsilesional myelination was found (p = 1.00).

## 4 Discussion

We used anatomical MRI to estimate differences in myelination between typically developing children and those with perinatal stroke, assessing both inter- and intra-hemispheric changes. Myelination patterns are altered after perinatal stroke. Differences in myelination appear to “radiate” outward from the lesion as the degree of myelination increased from perilesional to remote ipsilesional to contralesional hemisphere locations. That myelination of the contralesional hemisphere is altered following early unilateral brain injury suggests far-reaching effects on brain development not previously described in this population. Since our threshold methods assumed the contralesional hemisphere was normal, the amount of myelination in the affected hemisphere may have been underestimated given that myelination in the unaffected hemisphere was also affected.

The absence of significant associations between myelination measures and comprehensive outcome measures is clearly a statistical power issue due to the modest sample size of children who underwent formal neuropsychological testing. Specifically, some relationships between myelination ratios on certain tests had moderate effect sizes which is perhaps more informative than interpreting p-values in isolation. Given these effect sizes, a larger, more highly-powered sample may have been able to elucidate more clearly these relationships and reduce the probability of Type II errors (false negatives). Moderate effect sizes were found between WM myelination metrics and processing speed (WISC-IV PSI) as well as verbal learning (CVLT-C) and inhibition (NEPSY-II Inhibition-Switching) tasks all requiring rapid processing, speeded attention, and executive function. This pattern of results is consistent with that predicted given deficits in white matter myelination due to stroke. By contrast, FSIQ was not related to myelination possibly reflecting the relative insensitivity of this measure to subtle disruptions due to neurological disease. The absence of systematic relationships across testing domains supports the contribution of additional biological processes in the generation of the neurodevelopmental morbidities of perinatal stroke.

Aberrant myelination was most clear in the ipsilesional hemisphere and appeared to correlate directly with proximity to the primary lesion. This is perhaps not surprising given our primary hypothesis though the mechanisms by which this might occur are not immediately evident. White matter tracts proximate to arterial stroke lesions can certainly be abnormal as measured by diffusion imaging. The most established examples arise from studies of the corticospinal tracts where functionally relevant alterations in the acute (Groenendaal et al., 2006; Kirton et al., 2007a), subacute (van der Aa et al., 2013) and chronic (Hodge et al., 2017; Kuczynski et al., 2017a) timeframes have been shown. Recent evidence from acute diffusion weighted imaging studies in childhood stroke has also demonstrated diaschisis where diffuse alterations in white matter emanating out from stroke lesions toward connected structures can be observed (Kirton et al., 2016a, Kirton et al., 2016b). These acute imaging biomarkers may reflect the more diffuse disturbances inflicted by focal stroke lesions on more widely distributed white matter pathways.

That myelination appeared to be impaired in the contralesional hemisphere was an unexpected and very interesting discovery. That values were lower than both hemispheres in typically developing controls that were comparable in terms of both age and sex suggests significance. Non-motor morbidities are diverse and complex in children with perinatal stroke (Kirton and deVeber, 2013). Unlike motor impairments, the mechanisms by which they occur are poorly understood but are presumed to involve disruption of more diffuse neural networks, possibly including bilateral brain regions. Perhaps the best tangible example of how a unilateral early brain injury can influence global brain function is the increasingly described association between symptomatic seizures, epileptic encephalopathy, and adverse neuropsychological outcomes in this population (Mineyko et al., 2017; Trauner et al., 2013). This was the rationale for our exploration here that failed to demonstrate an association between epileptic encephalopathy and disordered myelination, though our power to do so was limited. While our findings require replication, the suggestion that unilateral strokes can influence bilateral brain development in some children warrants careful consideration and additional study.

Disturbances in myelination are established as a major determinant of adverse neurological outcomes in children born preterm (Back and Miller, 2014). Multiple risk factors appear to impart a risk of “developmental arrest” of early oligodendrocyte progenitors that often results in impaired white matter development, the degree of which correlates with developmental impairment. Such insults are incurred earlier in development than our arterial perinatal stroke population here, although another common form of perinatal stroke called fetal periventricular venous infarction (PVI) also injures the white matter prior to 34 weeks gestation (Kirton et al., 2008). White matter myelination certainly advances greatly between preterm and term timeframes but much continues beyond this, likely for decades. Therefore, although there are differences in both relative timing and focality between prematurity and perinatal stroke, our findings here of disordered myelination (including the contralesional hemisphere) suggest potential common ground for future study.

Did we really measure myelination? T1 signal intensity is certainly a valid biomarker of myelination in the developing brain. The earliest imaging signs of myelination in the preterm brain is an increase in white matter T1 signal (Hüppi and Dubois, 2006; Oishi et al., 2013; Welker and Patton, 2012). These evolving alterations of T1 signal are attributable to increasing amounts of myelin markers including cholesterol and galactocerebroside (Welker and Patton, 2012). Histological studies have further confirmed that myelin is the dominant source of MRI contrast in T1 maps (Stüber et al., 2014). Myelination delays have been found in neonatal rat models of hypoxia-ischemia, and are associated with long-term cognitive dysfunction (Huang et al., 2009; Segovia et al., 2008). These animal studies confirm alteration of myelination in ischemic injury, and validate the use of T1 signal as a measure of myelination. Studies in rat and mice models have shown strong correlations between myelin content and T1 signal (Harkins et al., 2016; Thiessen et al., 2013). This evidence suggests our use of T1 voxel intensity as a quantitative biomarker for myelination is valid but also not likely complete. Interpreting T1 differences between regions or subjects is also not necessarily reflective of differences in myelin content. Using a T1/T2 ratio may substantially improve the image contrast-to-noise ratio, potentially providing an improved differentiation between heavily and lightly myelinated areas (Glasser and Van Essen, 2011).

Our findings of significant asymmetry between hemispheres in typically developing controls further support the ability of our methods to detect differences in white matter signal. These findings are consistent with previous observations of increased fiber count in the major sensory white matter tracts of the non-dominant hemisphere in typically developing children (Kuczynski et al., 2017a, Kuczynski et al., 2017b). The mechanisms that mediate these differences are incompletely understood but increasingly informed by complementary approaches to white matter imaging in the developing brain (Lebel et al., 2017).

Emerging advanced MRI methods specifically designed to image myelin represent promising new directions. For example, a recent study of adults with chronic stroke and motor impairment applied a multi-component T2 relaxation imaging method to quantify myelin water fraction in functionally specific brain regions (Lakhani et al., 2017). The authors demonstrated that the degree of hemispheric asymmetry between the precentral regions correlated with clinical measures of disability. As this study was uncontrolled, the possibility of changes in the contralesional hemisphere could not be addressed (it was assumed to be normal), though perhaps this issue is more relevant to our developmental population. That such specific, clinically relevant changes in myelination can be demonstrated following stroke in the aged brain suggests potentially even more utility in the more dynamic developing brain after perinatal stroke or other neurodevelopmental conditions.

Our findings add another component to increasingly informed models of how the motor system develops following early unilateral brain injury. A resulting challenge is to then integrate these biomarkers with other measures of both white matter (as described above) but also grey matter where *FreeSurfer* can already segment and quantify regional cortical thickness, volume and surface area. Such anatomical approaches are further complemented by other advanced imaging approaches including MR spectroscopy and functional connectivity, which are demonstrating early evidence of applicability in perinatal stroke (Carlson et al., 2016; Ilvesmäki et al., 2017). Such personalized models are increasingly clinically relevant with recent translation into non-invasive neuromodulation clinical trials that suggest efficacy for improving function in hemiparetic children (Gillick et al., 2014; Kirton et al., 2016a, Kirton et al., 2016b; Kirton et al., 2017).

Important limitations are acknowledged. Given the diversity and complexity of outcomes from perinatal stroke (Kirton and Deveber, 2013), our study was likely underpowered to demonstrate associations between myelination and specific neurodevelopmental and motor deficits. Studies with similar comprehensive outcomes but with larger sample sizes will be required to better define the relationship between altered myelination and outcomes in perinatal stroke. As discussed above, we assumed that intensity of white matter on T1-weighted images is indicative of myelination. As T1-weighted MR imaging may be most useful during the first year of life (Welker and Patton, 2012), it may have provided a less complete picture of myelination in our older participants. Our motor assessments (AHA, Melbourne) were the best available, evidence-based measures but may only approximate real world function in children with hemiparesis. A large time interval was also sometimes present between clinical scans and motor assessments, further limiting our ability to establish clinical correlations.

## Declarations of interest

Brian Brooks receives royalties for the sales of three pediatric neuropsychological tests [Child and Adolescent Memory Profile (ChAMP, Sherman and Brooks, 2015, PAR Inc.), Memory Validity Profile (MVP, Sherman and Brooks, 2015, PAR Inc.), and Multidimensional Everyday Memory Ratings for Youth (MEMRY, Sherman and Brooks, 2017, PAR Inc.)].
